# Flexible Threshold Quantum Homomorphic Encryption on Quantum Networks

**DOI:** 10.3390/e27010007

**Published:** 2024-12-26

**Authors:** Yongli Tang, Menghao Guo, Binyong Li, Kaixin Geng, Jinxia Yu, Baodong Qin

**Affiliations:** 1 School of Software, Henan Polytechnic University, Jiaozuo 454000, China; 2 Advanced Cryptography and System Security Key Laboratory of Sichuan, Chengdu 610225, China; 3 School of Computer Science and Technology, Henan Polytechnic University, Jiaozuo 454003, China; 4 Shaanxi Key Laboratory of Information Communication Network and Security, Xi’an University of Posts & Telecommunications, Xi’an 710121, China

**Keywords:** threshold quantum homomorphic encryption, Shamir secret sharing, quantum computation, quantum computing cloud platform

## Abstract

Currently, most quantum homomorphic encryption (QHE) schemes only allow a single evaluator (server) to accomplish computation tasks on encrypted data shared by the data owner (user). In addition, the quantum computing capability of the evaluator and the scope of quantum computation it can perform are usually somewhat limited, which significantly reduces the flexibility of the scheme in quantum network environments. In this paper, we propose a novel (t,n)-threshold QHE (TQHE) network scheme based on the Shamir secret sharing protocol, which allows k(t≤k≤n) evaluators to collaboratively perform evaluation computation operations on each qubit within the shared encrypted sequence. Moreover, each evaluator, while possessing the ability to perform all single-qubit unitary operations, is able to perform arbitrary single-qubit gate computation task assigned by the data owner. We give a specific (3, 5)-threshold example, illustrating the scheme’s correctness and feasibility, and simulate it on IBM quantum computing cloud platform. Finally, it is shown that the scheme is secure by analyzing encryption/decryption private keys, ciphertext quantum state sequences during transmission, plaintext quantum state sequence, and the result after computations on the plaintext quantum state sequence.

## 1. Introduction

In the current era of information, safeguarding the security of private data is of paramount importance. Although traditional encryption techniques are excellent at securing data, the need to decrypt data arises when private data need to be computed and analyzed, which can potentially give rise to security risks. However, the privacy-preserving homomorphic encryption technique, as a revolutionary cryptographic tool, offers an innovative solution by allowing evaluation computations to be performed on encrypted data without the need for decryption. This means that the necessary computations and analyses can be performed while the private data remain encrypted, at the same time protecting the confidentiality and integrity of the private data. Since Rivest et al. [1] introduced the concept of classical fully homomorphic encryption (FHE) in 1978 and Gentry [2] proposed a classical FHE scheme in 2009, FHE has been widely used in various fields, including functional encryption [3], delegating computations [4], obfuscation [5] and plaintext encryption [6,7,8,9]. However, most classical FHE schemes cannot satisfy the security requirements of information theory.

With the rapid advancement in the field of cryptography, quantum cryptography has opened up a new avenue for the development of privacy-preserving homomorphic techniques. Quantum cryptography is based on the principles of quantum mechanics, and its security relies solely on the correctness of quantum mechanics, rather than computational assumptions. Therefore, it is capable of achieving information-theoretic security. In this context, quantum homomorphic encryption which integrates the concepts of homomorphic encryption and delegated quantum computation, as an important research branch in the field of quantum cryptography, provides a more secure data processing and computation mechanism, simultaneously offering a higher level of protection for sensitive information privacy.

In recent years, there has been a significant growth trend in research on quantum homomorphic encryption. This is attributed to the fact that quantum homomorphic encryption technology not only achieves the security requirements of information theory, but also provides an efficient approach to computing encrypted data. In 2012, Rohde et al. [10] proposed a restricted QHE scheme using the quantum wandering walk model. In 2013, Liang et al. [11] gave mathematical definitions of symmetric and asymmetric quantum homomorphic encryption schemes, and proposed a framework of QHE schemes with reference to classical homomorphic encryption schemes, which has been used until now. In their paper [11], four symmetric quantum homomorphic encryption schemes and one asymmetric quantum fully homomorphic encryption (QFHE) scheme were constructed based on the quantum one-time pad (QOTP). In 2015, Liang [12] constructed a QFHE scheme based on the quantum universal circuit. This scheme [12] uses the universal set of quantum gates {X,Y,Z,H,S,T,CNOT} to achieve arbitrary quantum computation. However, the decryption of a *T* gates in this scheme requires interaction steps and is only applicable to delegated quantum computation between two parties. In the same year, Broadbent and Jeffery [13] proposed two QHE schemes with a constant finite number of non-Clifford gates (such as *T* gates) in the quantum circuit, where evaluator can perform at most a constant number of non-Clifford gates on the encrypted data. They also give a formal definition of QFHE. In 2016, Dulek et al. [14] extended the research from the scheme [13] and introduced a novel QHE scheme, which can effectively compute quantum circuits of arbitrary polynomial size and correct errors that arise during the evaluation computation stage when computing the *T* gates on the ciphertext quantum state sequence. In 2018, Ouyang and Tan [15] proposed a QHE scheme with a constant number of non-Clifford gates based on quantum codes. In addition, numerous other QHE schemes based on various approaches have been introduced [16,17,18,19,20,21,22,23].

However, it should be highlighted that currently, most QHE schemes are primarily designed for scenarios involving only a single evaluator [10,11,12,13,14,15,17,18,19,20,21,22,23]. When a data owner requires an evaluator to perform a large number of evaluation computation tasks on his or her encrypted private data, the burden on the single evaluator becomes quite heavy and may not be able to respond in a timely manner to perform computation tasks assigned by other users [24,25,26,27]. The data owner sometimes may be unwilling to place complete trust in a single evaluator, instead, anticipate multiple evaluators collaborating to accomplish some significant evaluation computation tasks [28,29,30]. In addition, the flexibility of most QHE schemes is significantly diminished due to the constraints on the quantum computing capacity of the evaluator and the scope of quantum computation it can perform [12,13,15,23,31,32].

In 2019, Chen et al. [31] proposed a (t,n)-threshold QHE scheme with a flexible number of evaluators based on Cao and Ma’s [33](t,n)-threshold quantum state sharing (QSTS) scheme. In this scheme [31], the data owner selects any d(t≤d≤n) evaluators from the *n* evaluators to share encrypted data, and these *d* evaluators can collaboratively perform all evaluation computation tasks (single-qubit gate unitary operations) on the encrypted data. Then the data owner can obtain the expected result after computations on her private plaintext data by decrypting the final ciphertext data. However, the scheme proposed by Chen et al. [31] imposes restrictions on the types of single-qubit gate unitary operations that evaluators can perform on encrypted data. The first d−1 evaluators are limited to performing only single-qubit gate unitary operations from the set of single-qubit gates {X,Y,Z,H}, while the last evaluator is allowed to perform single-qubit gate unitary operations from the set of single-qubit gates {X,Y,Z,H,S,T}. That is, the first d−1 evaluators are not able to perform the single-qubit gates *S* and *T*, and not every evaluator can perform arbitrary single-qubit gate unitary operation in the set of single-qubit gates {X,Y,Z,H,S,T} on the encrypted data. We know that in the scheme [31], the *d* evaluators selected randomly from the *n* evaluators are a stochastic process. Since any evaluator could potentially be selected to serve as the *d*-th evaluator, each evaluator has the ability to perform single-qubit unitary operations {X,Y,Z,H,S,T,U(θ)}. However, it is important to note that the scope of quantum computation that evaluators can perform is subject to highly limitations.

In 2022, Liu et al. [32] proposed a (t,n)-threshold QHE scheme based on the Chen et al. [31] scheme. By exploring the relationship between the sets of single-qubit gates {X,Y,Z} and {H,S,T}, they transferred the operations of {H,S,T} which originally needed to be performed by the first d−1 evaluators to the last evaluator. It makes all *k* evaluators can perform any single-qubit gate unitary operation from the set of single-qubit gates $\left\{X,\;Y,\;Z,\;H,\;S,\;T\right\}$, but the first d−1 evaluators only have ability to perform the single-qubit unitary operations from $\left\{X,\;Y,\;Z,\;U(\theta )\right\}$, while the last evaluator have ability to perform the single-qubit unitary operations from $\left\{X,\;Y,\;Z,\;S,\;T,\;U(\theta )\right\}$. It also has a certain impact on the flexibility and efficiency of the scheme. For this we propose a novel TQHE scheme based on the Shamir (t,n)-threshold secret sharing protocol [34]. Our proposed scheme not only supports a flexible number of evaluators but also ensures that all evaluators have the ability to perform all single-qubit unitary operations from {X,Y,Z,H,S,T,U(θ)} and are allowed to perform any computation task assigned by the data owner on the encrypted data. The proposed TQHE scheme exhibits excellent flexibility and can be easily implemented through simple operations. In the future, we anticipate that this scheme will play a crucial role in quantum network communication applications, providing more solutions to address security and privacy concerns in the computation of private data.

### Our Contributions

First, we propose a novel (t,n)-threshold QHE scheme based on Shamir secret sharing, supporting any k(t≤k≤n) evaluators of the *n* evaluators to cooperatively perform homomorphic evaluation computations on the ciphertext quantum state sequence shared by the data owner Alice. Alice is able to obtain the expected result after computations on the plaintext quantum state sequence by decrypting the final computed ciphertext quantum state sequence. Second, in our TQHE scheme, all evaluators, while having the capability to perform all single-qubit unitary operations {X,Y,Z,H,S,T,U(θ)}, can perform any single-qubit gate unitary operation from the set of single-qubit gates {X,Y,Z,H,S,T} on the ciphertext quantum state sequence. Third, we provide a (3, 5)-threshold QHE example to clarify our scheme and validate its correctness and feasibility through simulations on the IBM quantum cloud platform. Finally, the security of the scheme is shown by analyzing the encryption/decryption private keys, ciphertext quantum state sequences during transmission, the plaintext quantum state sequence, and the result after computations on the plaintext quantum state sequence.

The remaining sections of this paper are organized as follows: Section 2 covers some preliminaries. Section 3 provides a detailed introduction to our novel TQHE scheme. Section 4 provides a concrete example and simulates it on the IBM quantum computing cloud platform. Section 5 analyzes the security. Section 6 provides some comparisons. Section 7 concludes the entire paper.

## 2. Preliminaries

In this section, we introduce some background knowledge that is crucial for understanding our scheme, including the classical Shamir (t,n)-threshold secret sharing protocol [34] and a brief overview of the TQHE scheme framework.

### 2.1. Shamir (t,n)-Threshold Secret Sharing Protocol

The principle of Shamir (t,n)-threshold secret sharing protocol consists of two core algorithms as follow.

(1) **The share generation algorithm:** Given a finite field GF(d), where *d* is a large prime number, the trusted data owner Alice randomly selects a t−1 degree polynomial:

(1) $$f\left(x\right)=a_{0}+a_{1}x+a_{2}x^{2}+\cdots +a_{t-1}x^{t-1}\;\mathrm{mod}\;d,$$

where $\left(a_{0},a_{1},a_{2},\ldots ,a_{t-1}\right)\in \mathrm{GF}\left(d\right)$, and $a_{0}$ is a secret integer. Alice selects *n* non-zero and distinct elements $\left\{x_{i}\in \mathrm{GF}\left(d\right)|i=\left(1,2,\ldots ,n\right)\right\}$ to compute *n* secret shares $\left\{f\left(x_{i}\right)\in \mathrm{GF}\left(d\right)|i=\left(1,2,\ldots ,n\right)\right\}$, and then securely transmits them to *n* participants $R=\left\{Bob_{g_{1}},Bob_{g_{2}},\cdots ,Bob_{g_{n}}\right\},g_{i}\in \left\{1,2,\ldots ,n\right\}\left(0<i\leq n\right)$ through secure classical channels, ensuring that each participant holds a private share $f\left(x_{i}\right)\in \mathrm{GF}\left(d\right)\left(i=1,2,\ldots ,n\right)$.

(2) **The secret reconstruction algorithm**: If any *t* participants $\left\{Bob_{1},Bob_{2},\cdots ,Bob_{t}\right\}$ out of *n* participants want to reconstruct the secret $a_{0}$, each participant $Bob_{i}$ in $\left\{Bob_{1},Bob_{2},\cdots ,Bob_{t}\right\}$ first takes out their private share $f(x_{i})$, and then uses the following Lagrange interpolation equation:

(2) $$f\left(x\right)=\left(\sum_{r=1}^{t}f\left(x_{r}\right)\prod_{1\leq j\leq t,j\neq r}\frac{x-x_{j}}{x_{r}-x_{j}}\right)\;\;\mathrm{mod}\;\;d,$$

to cooperate in reconstructing and calculating the secret information. In Equation (2), if x=0, then

(3) $$a_{0}=f\left(0\right)=\left(\sum_{r=1}^{t}f\left(x_{r}\right)\prod_{1\leq j\leq t,j\neq r}\frac{x_{j}}{x_{j}-x_{r}}\right)\;\mathrm{mod}\;d,$$

is the original secret integer.

In Equation (3), the secret information $a_{0}$ can be reconstructed by *t* participants using their private shares after cooperative computation. k(t≤k≤n) participants can still accurately reconstruct the secret information $a_{0}$ through cooperative computation using their private shares.

(4) $$\begin{array}{cc} a_{0} & =\left(\sum_{r=1}^{t}f\left(x_{r}\right)\prod_{1\leq j\leq t,j\neq r}\frac{x_{j}}{x_{j}-x_{r}}\right)\;\;\mathrm{mod}\;\;d\\ \; & =\left(\sum_{r=1}^{t+1}f\left(x_{r}\right)\prod_{1\leq j\leq t+1,j\neq r}\frac{x_{j}}{x_{j}-x_{r}}\right)\;\;\mathrm{mod}\;\;d\\ \; & =\cdots \\ \; & =\left(\sum_{r=1}^{k}f\left(x_{r}\right)\prod_{1\leq j\leq k,j\neq r}\frac{x_{j}}{x_{j}-x_{r}}\right)\;\;\mathrm{mod}\;\;d\\ \; & =\cdots \\ \; & =\left(\sum_{r=1}^{n}f\left(x_{r}\right)\prod_{1\leq j\leq n,j\neq r}\frac{x_{j}}{x_{j}-x_{r}}\right)\;\;\mathrm{mod}\;\;d.\; \end{array}$$

### 2.2. Definition of TQHE

Based on the existing TQHE schemes [31,32], we provide a specific definition for TQHE as follows.

**Definition 1**  (TQHE framework [31,32])**.** *A TQHE scheme consists of four main steps:*

*(1)* **Key Generation.** *$\mathrm{TQHE}.\mathrm{KeyGen}\;\left(1^{\kappa }\right)\to \left(sk_{e},\;\rho_{evk_{i}},\;sk_{d}\right)$. It is used to generate a series of keys, which include the encryption private key $sk_{e}$ and the decryption private key $sk_{d}$ for the data owner Alice, and evaluation keys $\rho_{evk_{i}}\;(i=1,2,\ldots ,k)$ for the k evaluators $\left\{Bob_{1},Bob_{2},\cdots ,Bob_{k}\right\}$;*

*(2)* **Encryption.** *$\mathrm{TQHE}.{\mathrm{Enc}}_{sk_{e}}(|m_{a}\rangle )\to (|c_{a}\rangle )$. Alice runs the $\mathrm{TQHE}.{\mathrm{Enc}}_{sk_{e}}(|m_{a}\rangle )$ to encrypt her original plaintext quantum state sequence $|m_{a}\rangle$ using her encryption key $sk_{e}$ to obtain the original ciphertext quantum state sequence $|c_{a}\rangle$;*

*(3)* **Homomorphic Evaluation.** *$\mathrm{TQHE}.\mathrm{Eval}\;(\chi_{i},\;\rho_{evk_{i}},\;\;\;|\;\;\;c_{a}\rangle )\to (|c_{b}\rangle )$. It is used to process the quantum ciphertext state sequence without decryption. After Alice shares the encrypted ciphertext quantum state sequence $|c_{a}\rangle$ to k(t≤k≤n) evaluators $\left\{Bob_{1},Bob_{2},\cdots ,Bob_{k}\right\}$, they run the $\mathrm{TQHE}.\mathrm{Eval}\;(\chi_{i},\;\rho_{evk_{i}},\;\;\;|\;\;\;c_{a}\rangle )$ sequentially to obtain the final homomorphic ciphertext quantum state sequence $|c_{f}\rangle$;*

*(4)* **Decryption.** *$\mathrm{TQHE}.{\mathrm{Dec}}_{sk_{d}}\;(\;\;\;|\;\;\;c_{f}\rangle )\to (|m_{f}\rangle )$. Alice runs the $\mathrm{TQHE}.{\mathrm{Dec}}_{sk_{d}}\;(\;\;\;|\;\;\;c_{f}\rangle )$ to decrypt the final ciphertext quantum state sequence $\;\;\;|\;\;\;c_{f}\rangle$ using her decryption key $sk_{d}$ to obtain the expected plaintext quantum state sequence $|m_{f}\rangle =\prod_{i=1}^{k}\chi_{i}|c_{a}\rangle$.*

*Here, $|m_{a}\rangle ,\;\;\;|\;\;\;m_{f}\rangle \in$ the quantum message space M, and $c_{a},c_{f}\in$ the quantum ciphertext space C. $\chi_{i}\in$ the set of quantum gates $F_{\;\;\;\Delta \;\;\;}$, i.e., $\chi_{i}\in F_{\;\;\;\Delta \;\;\;}$. As for the security of a TQHE scheme, it should satisfy indistinguishability under chosen-plaintext attacks (q-IND-CPA) in quantum polynomial time (QPT). Hence, a TQHE scheme is said to be q-IND-CPA secure if for any QPT adversary $A=(A_{1},A_{2})$ there exists a negligible function satisfying $\mathrm{Pr}\left[{\mathrm{PubK}}_{A,\mathrm{TQHE}}^{cpa}\left(\kappa \right)=1\right]\leq \frac{1}{2}+negl\left(\kappa \right)$, where ${\mathrm{PubK}}_{A,\mathrm{TQHE}}^{cpa}$ is a model of quantum indistinguishability under CPA [14].*

**Definition 2**  (Quantum indistinguishability under CPA)**.** *The game model of quantum indistinguishability under chosen-plaintext attack (IND-CPA) ${\mathrm{PubK}}_{A,\mathrm{TQHE}}^{cpa}\left(\kappa \right)$ for a TQHE scheme and a QPT adversary $A=(A_{1},A_{2})$ is defined as*

*(1) The challenger runs $\mathrm{TQHE}.\mathrm{KeyGen}\;\left(1^{\kappa }\right)\to \left(sk_{e},\;\rho_{evk_{j}},\;sk_{d}\right),j\in \left[1,k\right]\cap Z$.*

*(2) The challenger sends $\rho_{evk_{i}},i\in \left[2,k\right]\cap Z$ to $A_{1}$. Then, $A_{1}$ outputs a quantum state in M⊗E, where M is the message space and E is an arbitrary state related to the environment.*

*(3) For r∈{0,1}, let $\Xi_{\mathrm{TQHE}}^{cpa,r}:D\left(M\right)\to D\left(C\right)$ be $\Xi_{\mathrm{TQHE}}^{cpa,0}\left(\rho \right)=\mathrm{TQHE}.{\mathrm{Enc}}_{sk_{e}}(|0\rangle \langle 0|)$ and $\Xi_{\mathrm{TQHE}}^{\mathrm{cpa},1}\left(\rho \right)=\mathrm{TQHE}.{\mathrm{Enc}}_{sk_{e}}\left(\rho \right)$. A random bit r∈{0,1} is chosen and $\Xi_{\mathrm{TQHE}}^{cpa,0}\left(\rho \right)$ is applied to the state in M.*

*(4) $A_{2}$ obtains the state in C⊗E and outputs a bit $r^{\prime }$.*

*(5) The output of the game is defined as 1 if $r^{\prime }=r$ and 0 otherwise. If $r=r^{\prime }$, $A_{2}$ wins the game.*

## 3. Our Scheme

**Figure 1:**
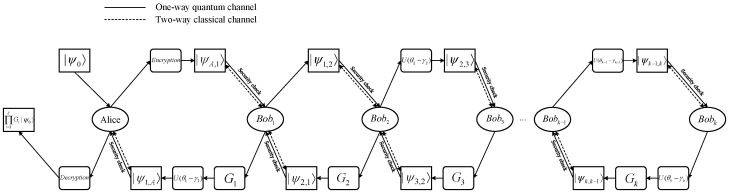
The main process of the $\left(t,\;n\right)$-threshold QHE scheme we proposed, where Encryption/Decryption refers to the encryption unitary operation $U\left(\theta_{A}\right)$ and decryption unitary operation $U(\gamma_{A}+\gamma_{1}+\gamma_{2}+\cdots +\gamma_{k})$, respectively.

In this section, we have proposed a novel TQHE scheme based on the Shamir (t,n)-threshold secret sharing protocol. It has a flexible number of evaluators, supporting k(t≤k≤n) evaluators to perform arbitrary single-qubit gate unitary operation (any evaluation computation task) from the set of single-qubit gates {X,Y,Z,H,S,T} on the ciphertext quantum state sequence. Figure 1 shows the main flow chart of the proposed scheme.

### The Proposed TQHE Scheme

In our TQHE scheme, initially, Alice encrypts her original plaintext quantum state sequence into the ciphertext quantum state sequence. Subsequently, k(t≤k≤n) evaluators sequentially perform the evaluation computation task on the ciphertext quantum state sequence encrypted by Alice. Then, the last evaluator sends the final ciphertext quantum state sequence to Alice. Finally, Alice decrypts it to obtain the result computed on the original plaintext quantum state sequence. The detailed procedure of our proposed TQHE scheme is given as follows.

**Step 1: Key generation stage**

In this stage, Alice executes the key generation algorithm [35] to generate a series of initial keys.

(1) **The shadow key generation sub-algorithm**

Initially, Alice runs the shadow key generation sub-algorithm to randomly create a polynomial of degree t−1, denoted as $f\left(x\right)=a_{0}+a_{1}x+a_{2}x^{2}+\cdots +a_{t-1}x^{t-1}\;\;\mathrm{mod}\;\;d,$ where GF(d) is a finite field, *d* is a randomly selected large prime number, and $\left(a_{0},a_{1},a_{2},\ldots ,a_{t-1}\right)$∈GF(d).

Afterwards, Alice randomly selects *n* different elements $\left\{x_{g_{i}}\in \mathrm{GF}\left(d\right)|g_{i}\in \left\{1,2,\ldots ,n\right\}\right\}$(0<i≤n) as inputs for the polynomial f(x) to sequentially obtain the shadow keys $\left\{wk_{g_{i}}=f\left(x_{i}\right)\in \mathrm{GF}\left(d\right)|i=\left(1,2,\ldots ,n\right)\right\}$ for each of the *n* participants $\left\{Bob_{g_{1}},Bob_{g_{2}},\ldots ,Bob_{g_{n}}\right\}$. Subsequently, Alice selects any k(t≤k≤n) participants $\left\{Bob_{1},Bob_{2},\ldots ,Bob_{k}\right\}$ from the group of the *n* participants $\left\{Bob_{g_{1}},Bob_{g_{2}},\ldots ,Bob_{g_{n}}\right\}$ as *k* evaluators, with $Bob_{1}$ being the first randomly chosen evaluator. Finally, Alice computes the private keys $\theta_{i}\left(i=1,2,\ldots ,k\right)$ of *k* evaluators according to Equations (5) and (6), where

(5) $$\theta_{i}=2\pi \cdot \frac{L_{i}wk_{i}}{d}\;\mathrm{mod}\;d,$$

(6) $$L_{i}=\prod_{1\leq r\leq k,r\neq i}\frac{x_{r}}{x_{r}-x_{i}}\mathrm{mod}\;d.$$

The private key $\theta_{1}$ of $Bob_{1}$ is kept secretly by Alice, while the remaining k−1 private keys $\left\{\theta_{2},\theta_{3},\ldots ,\theta_{k}\right\}$ of $\left\{Bob_{2},Bob_{3}\ldots ,Bob_{k}\right\}$ are secretly distributed by Alice over secure classical channels.

(2) **The rotation key generation sub-algorithm**

Alice runs the rotation key generation sub-algorithm to generate *k* randomly different rotation keys $\left\{\gamma_{i}\in \left[0,2\pi \right]|i=1,2,\ldots ,k\right\}$ and then distributes the k−1 rotation keys $\left\{\gamma_{2},\gamma_{3},\ldots ,\gamma_{k}\right\}$ to the k−1 evaluators $\left\{Bob_{2},Bob_{3},\ldots ,Bob_{k}\right\}$ through secure classical channels. Similarly, the rotation key $\gamma_{1}$ of $Bob_{1}$ is kept secretly by Alice.

**Step 2: Encryption sharing stage**

(1) The data owner Alice has an original $|\psi_{0}\rangle$ plaintext quantum state sequence of length *m*:

(7) $$|\psi_{0}\rangle =\{|{\varphi_{0}}_{,u}\rangle =\alpha_{0,u}|0\rangle +\beta_{0,u}|1\rangle |u=1,2,\ldots ,m\},$$

where $|\alpha_{0,u}{|}^{2}+|\beta_{0,u}{|}^{2}=1$. She defines a 2×2 unitary matrix $U\left(\theta \right)\;=\left(\begin{array}{cc} \cos(\theta ) & -\sin(\theta )\\ \sin(\theta ) & \cos(\theta ) \end{array}\right)$, and an angle parameter “θ” is introduced to describe a specific transformation or operation of the matrix. The behavior of this matrix will depend on the specific value of “θ”. U(θ) can be used to achieve various types of qubit operations, such as phase shift transformation of the quantum state. Therefore, by adjusting “θ”, we can perform different phase shift unitary operations on the quantum state, which in turn enables the protection of private data. Performing the phase shift unitary operation U(θ) on a qubit state |φ〉=α|0〉+β|1〉 satisfies Equation (8):

(8) $$U\left(\theta_{1}\right)U\left(\theta_{2}\right)|\varphi \rangle =U\left(\theta_{1}+\theta_{2}\right)|\varphi \rangle =\left(\begin{array}{cc} \cos(\theta_{1}+\theta_{2}) & -\sin(\theta_{1}+\theta_{2})\\ \sin(\theta_{1}+\theta_{2}) & \cos(\theta_{1}+\theta_{2}) \end{array}\right)|\varphi \rangle .$$

(2) Alice randomly selects c∈Z and uses $\theta_{A}=2\pi -2\pi \cdot \frac{a_{0}}{d}-\gamma_{A}$ as her encryption private key where $\gamma_{A}=2c\pi -\theta_{1}-\gamma_{2}-\gamma_{3}-\cdots -\gamma_{k}$, $\theta_{1}=2\pi \cdot \frac{L_{1}wk_{1}}{d}\;\mathrm{mod}\;d$ and $L_{1}=\prod_{1\leq r\leq k,r\neq 1}\frac{x_{r}}{x_{r}-x_{1}}\;\mathrm{mod}\;\;d$. Next, she performs the encryption unitary operation $U(\theta_{A})$ on the plaintext quantum state sequence $|\psi_{0}\rangle$, resulting in the original encrypted ciphertext quantum state sequence

(9) $$\begin{array}{cc} |\psi_{A,1}\rangle & =U\left(\theta_{A}\right)|\psi_{0}\rangle \\ \; & =U\left(\theta_{A}\right)\{|\varphi_{0,1}\rangle ,|\varphi_{0,2}\rangle ,\ldots ,|\varphi_{0,m}\rangle \}\\ \; & =\{|\varphi_{A,1,1}\rangle ,|\varphi_{A,1,2}\rangle ,\ldots ,|\varphi_{A,1,3}\rangle \}. \end{array}$$

(3) Alice randomly prepares some decoy particles from states $\{|0\rangle ,|1\rangle ,|+\rangle =(|0\rangle +|1\rangle )/\sqrt{2},|-\rangle =(|0\rangle -|1\rangle )/\sqrt{2}\}$, randomly inserts these decoy particles into the ciphertext quantum state sequence $|\psi_{A,1}\rangle$ to obtain a new quantum state sequence ${|\psi_{A,1}\rangle }^{\prime }$, and meticulously records the insertion positions and initial states of each decoy particle. These decoy particles are primarily used for the security check during the transmission of quantum ciphertext sequences [36]. Subsequently, Alice transmits ${|\psi_{A,1}\rangle }^{\prime }$ to the first evaluator $Bob_{1}$ over a quantum channel. After confirming that $Bob_{1}$ has received ${|\psi_{A,1}\rangle }^{\prime }$, Alice informs $Bob_{1}$ of the positions where each decoy particle was inserted and the corresponding measurement basis (X-basis or Z-basis). Afterwards, $Bob_{1}$ measures the states of these decoy particles using the corresponding basis (X-basis or Z-basis) and sends the measurement results to Alice. Alice compares whether the results from $Bob_{1}$ match the initially states of the decoy particles. If the error rate is below a certain low threshold value, Alice instructs $Bob_{1}$ to proceed to the next step; otherwise, Alice will ask $Bob_{1}$ to abort the process and restart a new one. It is important to note that, during each transmission of the quantum state sequence, both the sender and the receiver should conduct similar security check procedures and this process will not be described repeatedly in the following text.

(4) Following the successful security check, $Bob_{1}$ removes the decoy particles from the sequence ${|\psi_{A,1}\rangle }^{\prime }$ to obtain the ciphertext quantum state sequence $|\psi_{A,1}\rangle$. During this stage, $Bob_{1}$ temporarily refrains from performing the evaluation computation operation and the phase shift unitary operation on the sequence $|\psi_{A,1}\rangle$. Instead, he randomly re-inserts decoy particles into the sequence $|\psi_{A,1}\rangle$ to obtain a new quantum state sequence ${|\psi_{1,2}\rangle }^{\prime }$ and then transmits it to the next evaluator $Bob_{2}$ through a quantum channel.

(5) After receiving ${|\psi_{1,2}\rangle }^{\prime }$ on security, $Bob_{2}$ removes the decoy particles to obtain the quantum state sequence $|\psi_{1,2}\rangle =|\psi_{A,1}\rangle$. Then, $Bob_{2}$ applies the phase shift unitary operation $U(\theta_{2}-\gamma_{2})$ on each individual qubit in the quantum state sequence $|\psi_{1,2}\rangle$, resulting in the quantum state sequence

(10) $$|\psi_{2,3}\rangle =U\left(\theta_{2}-\gamma_{2}\right)|\psi_{1,2}\rangle .$$

Next, $Bob_{2}$ sends ${|\psi_{2,3}\rangle }^{\prime }$ formed by inserted decoy particles into $|\psi_{2,3}\rangle$ to the next evaluator $Bob_{3}$. Similarly, $Bob_{3},Bob_{4},\ldots ,Bob_{k-1}$ perform the security check and the phase shift operation $U\left(\theta_{3}-\gamma_{3}\right),U\left(\theta_{4}-\gamma_{4}\right),\ldots ,U\left(\theta_{k-1}-\gamma_{k-1}\right)$ on their respective received quantum state sequences $|\psi_{2,3}\rangle ,|\psi_{3,4}\rangle ,\ldots ,|\psi_{k-2,k-1}\rangle$. Finally, $Bob_{k-1}$ sends the quantum state sequence ${|\psi_{k-1,k}\rangle }^{\prime }$ with inserted decoy particles to the *k*-th evaluator $Bob_{k}$.

**Step 3: Evaluation computation stage**

In this stage, Alice sends the order in which k(t≤k≤n) evaluators perform calculations to them separately through a secure classical channel. Moreover, Alice divides the evaluation computation tasks $G=G_{1}G_{2}\cdots G_{k}$ that she wishes to perform on the original plaintext quantum state sequence into *k* segments, and distributes the segmented task $G_{i}=\prod_{v=1}^{p}V_{v},\;(V_{v}\in \left\{X,Y,Z,H,S,T\right\},p\in Z_{+},\;\mathrm{and}\;i=1,2,\ldots ,k)$ to the *i*-th evaluator $Bob_{i}$ over secure classical channels for the subsequent evaluation computation. In our scheme, the single-qubit gate unitary operation assigned to the evaluators comes from the set of single-qubit gates {X,Y,Z,H,S,T}, where $X=\left(\begin{array}{cc} 0 & 1\\ 1 & 0 \end{array}\right),Y=\left(\begin{array}{cc} 0 & -i\\ i & 0 \end{array}\right),Z=\left(\begin{array}{cc} 1 & 0\\ 0 & -1 \end{array}\right),H=\frac{1}{\sqrt{2}}\left(\begin{array}{cc} 1 & 1\\ 1 & -1 \end{array}\right),S=\left(\begin{array}{cc} 1 & 0\\ 0 & i \end{array}\right)\;\mathrm{and}\;T=\left(\begin{array}{cc} 1 & 0\\ 0 & e^{\pi i/4} \end{array}\right)$. The set {X,Y,Z,H,S,T} is the universal set of single-qubit gates. These single-qubit gates can be used to approximate any single-qubit unitary operation with arbitrary precision [37]. A quantum state of a single qubit can be represented as |φ〉=α|0〉+β|1〉, where α and β are complex numbers satisfying $|\alpha \rangle^{2}+|\beta \rangle^{2}=1$. These universal single-qubit gates are used to manipulate individual qubits to implement an arbitrary single-qubit transformation in the evaluation computation stage. Evaluators can utilize these gates to rotate and transform the state of qubits, enabling the execution of various quantum evaluation computation tasks. Our scheme involves performing the evaluation computation tasks *G* on a sequence of quantum states. This is achieved by performing single-qubit gate unitary operations from the set of single-qubit gates {X,Y,Z,H,S,T} on each qubit of the quantum state sequence. The *k* evaluators sequentially perform the evaluation computation task $G_{i}\;\left(0<i\leq k\right)$ (the composite single-qubit gate unitary operation) assigned to them by Alice, and $Bob_{1}$ finally sends the final ciphertext quantum state sequence after completing the evaluation computation tasks for Alice.

(1) After the quantum state sequence ${|\psi_{k-1,k}\rangle }^{\prime }$ successfully passes the security check, $Bob_{k}$ removes the decoy particles to obtain the quantum state sequence $|\psi_{k-1,k}\rangle$. Next, he sequentially applies the phase shift unitary operation $U(\theta_{k}-\gamma_{k})$ and performs the evaluation computation operation $G_{k}$ on the sequence $|\psi_{k-1,k}\rangle$ to obtain the quantum state sequence

(11) $$|\psi_{k,k-1}\rangle =G_{k}U\left(\theta_{k}-\gamma_{k}\right)|\psi_{k-1,k}\rangle .$$

$Bob_{k}$ then sends ${|\psi_{k,k-1}\rangle }^{\prime }$ obtained after inserting the decoy particles in $|\psi_{k,k-1}\rangle$ to the next evaluator $Bob_{k-1}$.

(2) Following the successful security check, $Bob_{k-1}$ performs the evaluation computation operation $G_{k-1}$ on the received sequence $|\psi_{k,k-1}\rangle$ and obtains the quantum state sequence

(12) $$|\psi_{k-1,k-2}\rangle =G_{k-1}|\psi_{k,k-1}\rangle .$$

$Bob_{k-1}$ then sends ${|\psi_{k-1,k-2}\rangle }^{\prime }$ formed by inserted decoy particles into $|\psi_{k-1,k-2}\rangle$ to the next evaluator $Bob_{k-2}$.

(3) $Bob_{k-2},Bob_{k-3},\ldots ,Bob_{2}$ do something similar to $Bob_{k-1}$. After successfully passing the security check, they perform the evaluation computation operation $G_{k-2},G_{k-3},\cdots ,G_{2}$ on the received sequence $|\psi_{k-1,k-2}\rangle ,|\psi_{k-2,k-3}\rangle ,\ldots ,|\psi_{3,2}\rangle$, respectively. In the end, $Bob_{2}$ sends ${|\psi_{2,1}\rangle }^{\prime }$ obtained after inserting the decoy particles in $|\psi_{2,1}\rangle$ to the next evaluator $Bob_{1}$.

(4) After passing the successful security check, $Bob_{1}$ sequentially performs the evaluation computation operation $G_{1}$ and the phase shift unitary operation U(σ) ($\sigma =\theta_{1}-\gamma_{1}$ is secretly calculated by Alice and then secretly shared with $Bob_{1}$, who has no knowledge of the private key $\theta_{1}$ and the rotation key $\gamma_{1}$) on the sequence $|\psi_{2,1}\rangle$, resulting in the quantum state sequence

(13) $$|\psi_{1,A}\rangle =U\left(\sigma \right)G_{1}|\psi_{2,1}\rangle .$$

Finally, $Bob_{1}$ sends ${|\psi_{1,A}\rangle }^{\prime }$ obtained after inserting the decoy particles in $|\psi_{1,A}\rangle$ to the data owner Alice.

**Step 4: Decryption stage**

After securely receiving $|\psi_{1,A}\rangle$ resulting from the collaborative computations of k(t≤k≤n) evaluators, sent by $Bob_{1}$ who performed the last evaluation computation operation, Alice uses her rotation key $\gamma_{A}$ and the random rotation keys $\left\{\gamma_{i}|i=1,2,\ldots ,k\right\}$ of the *k* evaluators to perform the decryption unitary operation $D=U(\gamma_{A}+\gamma_{1}+\gamma_{2}+\cdots +\gamma_{k})$ on the sequence $|\psi_{1,A}\rangle$. Taking the *u*-th quantum bit $|\varphi_{1,A,u}\rangle$ of the decryption sequence $|\psi_{1,A}\rangle$ as an example, the decryption process is illustrated below:

(14) $$\begin{array}{cc} \; & D|\varphi_{1,A,u}\rangle =U\left(\gamma_{A}+\gamma_{1}+\gamma_{2}+\cdots +\gamma_{k}\right)|\varphi_{1,A,u}\rangle \\ \; & =U\left(\gamma_{A}+\gamma_{1}+\gamma_{2}+\cdots +\gamma_{k}\right)U\left(\theta_{1}-\gamma_{1}\right)G_{1}G_{2}\cdots G_{k}U\left(\theta_{k}-\gamma_{k}\right)\cdots U\left(\theta_{2}-\gamma_{2}\right)|\varphi_{1,A,u}\rangle \\ \; & =G_{1}G_{2}\cdots G_{k}U\left(\gamma_{A}+\gamma_{1}+\gamma_{2}+\cdots +\gamma_{k}\right)U\left(\theta_{1}-\gamma_{1}\right)U\left(\theta_{k}-\gamma_{k}\right)\cdots U\left(\theta_{2}-\gamma_{2}\right)U\left(\theta_{A}\right)|\varphi_{0,u}\rangle \\ \; & =G_{1}G_{2}\cdots G_{k}U\left(\gamma_{A}+\theta_{A}+\frac{2\pi }{d}\cdot a_{0}\right)|\varphi_{0,u}\rangle \\ \; & =\prod_{i=1}^{k}G_{i}U\left(2\pi \right)|\varphi_{0,u}\rangle \\ \; & =\prod_{i=1}^{k}G_{i}|\varphi_{0,u}\rangle . \end{array}$$

In the above Equation (14), it should be noted that $U\left(\gamma_{A}+\gamma_{1}+\gamma_{2}+\cdots +\gamma_{k}\right)U\left(\theta_{1}-\gamma_{1}\right)=U\left(\gamma_{A}+\theta_{1}+\gamma_{2}+\cdots +\gamma_{k}\right)=I$. Apparently, Alice performs the decryption unitary operation on the final ciphertext quantum state sequence $|\psi_{1,A}\rangle$ and obtains the correct result, which is the same as that obtained by performing evaluation computation directly on the plaintext quantum state sequence $|\psi_{0}\rangle$:

(15) $$D|\psi_{1,A}\rangle =\prod_{i=1}^{k}G_{i}|\psi_{0}\rangle .$$

Specifically, any k(t≤k≤n) evaluators selected from *n* evaluators sequentially perform the evaluation computation operation on the ciphertext quantum state sequence encrypted by data owner Alice. The last evaluator, after completing the collaborative computations, sends the final ciphertext quantum state sequence to Alice. Alice decrypts it to obtain the expected result of computing on the plaintext quantum state sequence. In other words, the result of Alice’s decryption operation on the final ciphertext quantum state sequence is the same as the result of Alice performing the same evaluation computation operations directly on the plaintext quantum state sequence.

## 4. Example and Simulation

**Figure 2:**
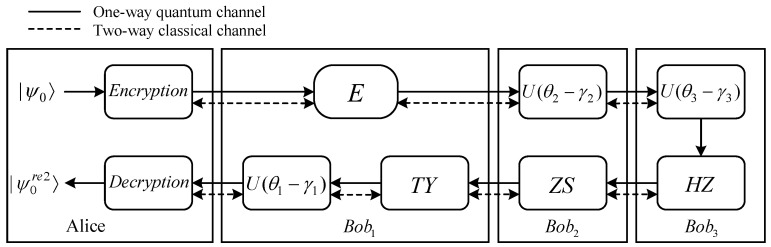
The example process of (3, 5)-threshold QHE for the proposed scheme, where $Encryption=U(\theta_{A})$, $Decryption=U(\gamma_{A}+\gamma_{1}+\gamma_{2}+\gamma_{3})$, and *E* indicates that no evaluation computation operation and unitary operation are being performed.

In Section 3, we proposed a novel TQHE scheme and analyzed its correctness. To better illustrate the scheme, in this section, we first present a specific example of (3, 5)-threshold QHE to demonstrate the correctness and feasibility of the TQHE scheme (Figure 2). Subsequently, the correctness of the example is verified by running simulation experiments on the IBM quantum computing cloud platform.

### 4.1. Example

In this example, suppose Alice wants at least three evaluators to collaborate on the evaluation computation tasks *G*. First, Alice selects three participants $\left\{Bob_{1},Bob_{2},Bob_{3}\right\}$ out of the five participants $\left\{Bob_{g_{1}},Bob_{g_{2}},Bob_{g_{3}},Bob_{g_{4}},Bob_{g_{5}}\right\}$ as evaluators to perform the evaluation computation operations on Alice’s encrypted quantum state sequence $|\psi_{A,1}\rangle$. After completing the collaborative computations, the last evaluator $Bob_{1}$ sends the final ciphertext quantum state sequence $|\psi_{1,A}\rangle$ to Alice. Finally, Alice can obtain the expectant result by decrypting $|\psi_{1,A}\rangle$. In the following example, the security check steps are excluded.

First, Alice generates a series of keys. By inputting parameters n=5,t=3,d=7 into the shadow key generation sub-algorithm, she obtains a random quadratic polynomial $f\left(x\right)=\left(2+3x+x^{2}\right)\;\mathrm{mod}\;7$ with coefficients $\left\{a_{0}=2,a_{1}=3,a_{2}=1\right\}$, where $a_{0}$ is the secret information. Next, by inputting five parameters $\left\{x_{g_{1}}=1,x_{g_{2}}=3,x_{g_{3}}=5,x_{g_{4}}=2,x_{g_{5}}=4\right\}$ into the polynomial f(x), she obtains five shadow keys $\left\{wk_{g_{1}}=f\left(x_{g_{1}}\right)=f\left(1\right)=6,wk_{g_{2}}=f\left(3\right)=6,wk_{g_{3}}=f\left(5\right)=0,wk_{g_{4}}=f\left(2\right)=5,wk_{g_{5}}=f\left(4\right)=2\right\}$.

Suppose Alice selects three evaluators $\left\{Bob_{1},Bob_{2},Bob_{3}\right\}$ to perform the evaluation computation operation tasks G=TYZSHZ on the original plaintext quantum state sequence $|\psi_{0}\rangle =\frac{\sqrt{2}}{2}(|0\rangle +\left|1\rangle \right)$, which consists of only a single quantum bit.

First, Alice computes the private keys $\{\theta_{1}=2\pi \cdot \frac{L_{1}wk_{1}}{d}=2\pi \cdot \frac{1}{d}\left(\left(f\left(x_{1}\right)\frac{x_{2}\cdot x_{3}}{\left(x_{2}-x_{1}\right)\left(x_{3}-x_{1}\right)}\right)\;\mathrm{mod}\;\;d\right)$=$\frac{8}{7}\pi ,\theta_{2}=\frac{12}{7}\pi ,\theta_{3}=\frac{12}{7}\pi \}\left(x_{1}=x_{g_{1}}=1,x_{2}=x_{g_{4}}=2,x_{3}=x_{g_{2}}=3;wk_{2}=wk_{g_{4}}=f\left(x_{g_{4}}\right)=5,wk_{3}=wk_{g_{2}}=f\left(x_{g_{2}}\right)=6\right)$ and generates the rotation keys $\left\{\gamma_{1}=\frac{17}{21}\pi ,\gamma_{2}=\frac{55}{28}\pi ,\gamma_{3}=\frac{65}{42}\pi \right\}$ by running the rotation key generation sub-algorithm. Later, Alice communicates with $\left\{Bob_{1},Bob_{2},Bob_{3}\right\}$ to instruct them to perform the evaluation computation operation $\left\{G_{1}=TY,G_{2}=ZS,G_{3}=HZ\right\}$, and secretly distributes their respective shadow keys $\left\{\theta_{2},\theta_{3}\right\}$ and rotation keys $\left\{\gamma_{2},\gamma_{3}\right\}$ to the evaluators $\left\{Bob_{2},Bob_{3}\right\}$ through secure classical channels. $Bob_{1}$’s private key $\theta_{1}$ and rotation key $\gamma_{1}$ are kept secretly by Alice.

Next, Alice utilizes her encryption private key $\theta_{A}=2\pi -2\pi \cdot \frac{a_{0}}{d}-\gamma_{A}=\frac{343}{84}\pi \;\left(\gamma_{A}=2\pi -\theta_{1}-\gamma_{2}-\gamma_{3}=-\frac{223}{84}\pi \right)$ to perform the encryption unitary operation $U(\theta_{A})$ on the plaintext quantum state sequence $|\psi_{0}\rangle$ and obtains the encrypted quantum state sequence

(16) $$|\psi_{A,1}\rangle =U\left(\theta_{A}\right)|\psi_{0}\rangle \;=\frac{1}{2}|0\rangle +\frac{\sqrt{3}}{2}|1\rangle ,$$

and then transmits it to $Bob_{1}$.

After receiving $|\psi_{A,1}\rangle$, instead of performing the evaluation computation operation and the phase shift unitary operation on it, $Bob_{1}$ directly transmits $|\psi_{A,1}\rangle$ (renamed to $|\psi_{1,2}\rangle$) to $Bob_{2}$.

After receiving $|\psi_{1,2}\rangle$, $Bob_{2}$ applies the phase shift unitary operation $U(\theta_{2}-\gamma_{2})$ on $|\psi_{1,2}\rangle$, yielding

(17) $$|\psi_{2,3}\rangle =U\left(\theta_{2}-\gamma_{2}\right)|\psi_{1,2}\rangle =\frac{\sqrt{2}+\sqrt{6}}{4}|0\rangle +\frac{\sqrt{6}-\sqrt{2}}{4}|1\rangle ,$$

and then sends the quantum state sequence $|\psi_{2,3}\rangle$ to $Bob_{3}$.

After receiving $|\psi_{3,2}\rangle$, $Bob_{2}$ performs the evaluation computation operation $G_{2}$ on $|\psi_{3,2}\rangle$, yielding

(18) $$|\psi_{2,1}\rangle =G_{2}|\psi_{3,2}\rangle =-i|1\rangle ,$$

and then sends the quantum state sequence $|\psi_{2,1}\rangle$ to $Bob_{1}$.

After receiving $|\psi_{2,1}\rangle$, $Bob_{1}$ sequentially performs the evaluation computation operation $G_{1}$ and the phase shift unitary operation U(σ) ($\sigma =\theta_{1}-\gamma_{1}$ is secretly calculated by Alice and then secretly shared with $Bob_{1}$) on $|\psi_{2,1}\rangle$, yielding

(19) $$|\psi_{1,A}\rangle =U\left(\sigma \right)G_{1}|\psi_{2,1}\rangle =-\frac{1}{2}|0\rangle -\frac{\sqrt{3}}{2}|1\rangle ,$$

and then sends the quantum state sequence $|\psi_{1,A}\rangle$ to Alice.

Finally, Alice performs the decryption unitary operation $D=U(\gamma_{A}+\gamma_{1}+\gamma_{2}+\gamma_{3})$ on the quantum state sequence $|\psi_{1,A}\rangle$ resulting from the cooperative evaluation computation operations by the three evaluators, and obtains the final result

(20) $$|\psi_{0}^{re2}\rangle =D|\psi_{1,A}\rangle =U\left(\gamma_{A}+\gamma_{1}+\gamma_{2}+\gamma_{3}\right)|\psi_{1,A}\rangle =-|0\rangle .$$

Now, let us analyze the result of directly using the evaluation computation operator *G* on the original plaintext quantum state sequence $|\psi_{0}\rangle$. Obviously, the result is

(21) $$|\psi_{0}^{re1}\rangle =G|\psi_{0}\rangle =TYZSHZ|\psi_{0}\rangle =-|0\rangle .$$

From here, we see that Alice’s decrypted result $|\psi_{0}^{re2}\rangle$ is the same as the result $|\psi_{0}^{re1}\rangle$ obtained by direct computation on the plaintext quantum state. Theoretically, if Alice measures $|\psi_{0}^{re2}\rangle$ in Z-basis, she can obtain a measurement result of 0 with the probability 100%.

### 4.2. Simulation Experiment

**Figure 3:**
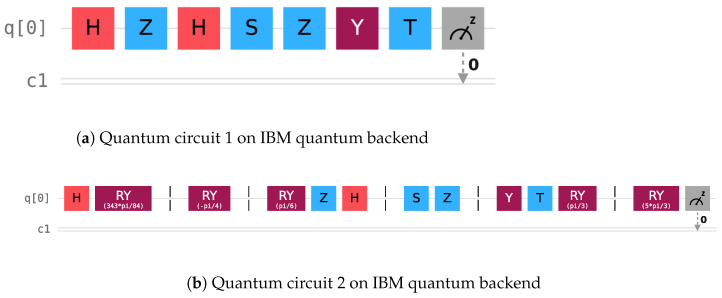
The quantum circuit diagram of the composite single-qubit gates TYZSHZ directly acting on $|\psi_{0}\rangle$ in the IBM quantum computing cloud platform in the subplot (**a**). The quantum circuit diagram of the (3, 5)-threshold QHE example in the IBM quantum computing cloud platform in the subplot (**b**), where RY(θ) represents the phase shift unitary operation U(θ).

On the IBM quantum computing cloud platform, we experimentally verify the correctness and feasibility of the given (3, 5)-threshold QHE example. The quantum circuit diagram for this example is shown in Figure 3b. Considering that the IBM quantum computing cloud platform does not allow operations to be performed on an arbitrary quantum state (the initial quantum state of a single quantum circuit in the IBM quantum computing cloud platform is the |0〉 state) and has some limitations on the number and spatial dimensions of quantum states [38,39,40], the experimental verification process of inserting and removing decoy particles is omitted in this section.

Five rounds of experiments are conducted on the (3, 5)-threshold example using two different backends, ‘ibm_brisbane’ and ‘ibm_lagos’, and three different measurement shots, 1024, 4096 and 8192, for the quantum circuit diagram presented in Figure 3b. The experimental measurement results are shown in Table 1.

On the one hand, on the quantum computing cloud platform, we measure the results of the composite evaluation computation operations $G_{1}G_{2}G_{3}$ directly acting on Alice’s original plaintext quantum state $|\psi_{0}\rangle$. The quantum circuit diagram is shown in Figure 3a.

On the other hand, we simulate the entire process of the (3, 5)-threshold QHE example on the quantum computing cloud platform and measure the results of performing all the evaluation computation operations and the unitary operations. The quantum circuit diagram is shown in Figure 3b.

The results of the 1024 measurements performed on the backend ‘ibm_brisbane’ and ‘ibm_logo’ after applying the composite single quantum bit gate $G=G_{1}G_{2}G_{3}$ directly to the original quantum plaintext quantum state $|\psi_{0}\rangle =\frac{\sqrt{2}}{2}(|0\rangle +\left|1\rangle \right)$ have a high probability (99.2% and 100%) corresponding to the state |0〉, respectively.

In all the measurement results in Table 1, the results of the 8192 measurements performed on the backend ‘ibm_brisbane’ have the smallest probability (99.2%) corresponding to the state |0〉, which shows that the fidelity should be the smallest in this situation. Assuming the theoretical density matrix is denoted by $\rho^{T}=1.0|0\rangle \langle 0|+0.0|1\rangle \langle 1|$, and the experimental density matrix is denoted by $\rho^{E}=0.992|0\rangle \langle 0|+0.008|1\rangle \langle 1|$, the smallest fidelity can be calculated as follows:

(22) $$F\left(\rho^{T},\rho^{E}\right)=Tr\sqrt{\sqrt{\rho^{T}}\cdot \rho^{E}\cdot \sqrt{\rho^{T}}}=99.5\%,$$

the fidelity of 99.5% is very close to the theoretical value of 100%. In the five rounds of experiments, the 99.5% fidelity is the lowest, and the fidelities of the other twenty-nine measurement results are higher than the fidelity of this particular measurement. By calculation, the average fidelity of the thirty experiments is 99.8%, which implies that all the obtained fidelity values from the measurements are very close to the theoretical value of 100%. Thus, the experimental results further validate the correctness of the (3, 5)-threshold QHE example and the feasibility of the proposed TQHE scheme.

## 5. Security Analysis

In this section, we analyze the security of the proposed TQHE scheme in three aspects. The analysis includes the security of the encryption/decryption private keys, ciphertext quantum state sequences during transmission, the plaintext quantum state sequence, and the result after computations on the plaintext quantum state sequence.

(1) **The security of the encryption/decryption private keys**

**Theorem 1.**  *The proposed TQHE scheme is perfect with respect to the probability distribution of the encryption private key $\theta_{e}$ over the private key space, that is,*

(23) $$I\left(\theta_{e};\;\;\;\Omega \;\;\;\right)=H\left(\theta_{e}\right)-H\left(\theta_{e}|\;\;\;\Omega \;\;\;\right)=0,$$

*where *Ω* denotes the set of key information distributed to the evaluators, $H(\theta_{e})$ is the information entropy of the encryption private key $\theta_{e}$ and $I\left(\theta_{e};\;\;\;\Omega \;\;\;\right)$ represents the mutual information of $\theta_{e}$ with *Ω*.*

**Proof.**  We know Alice’s encryption private key $\theta_{e}=2\left(c-1\right)\pi -2\pi \cdot \frac{a_{0}}{d}+\theta_{1}+\gamma_{2}+\gamma_{3}+\cdots +\gamma_{k}$. Essentially, the private share $\theta_{1}$ in $\theta_{e}$, and the rotation keys $\left\{\gamma_{2},\gamma_{3},\ldots ,\gamma_{k}\right\}$ are each derived independently from a uniform distribution, and since $\theta_{1}$ is kept secret by Alice, the secret $a_{0}$ cannot be recovered even if all evaluators conspire [34], which provides the maximum entropy for the encryption key. The conditional entropy of the encryption key $\theta_{e}$ is the same as its total entropy, $H\left(\theta_{e}|\;\;\;\Omega \;\;\;\right)=H\left(\theta_{e}\right)$, and hence the mutual information $I\left(\theta_{e};\;\;\;\Omega \;\;\;\right)=H\left(\theta_{e}\right)-H\left(\theta_{e}|\;\;\;\Omega \;\;\;\right)=0$, proving the security of the encryption key $\theta_{e}$ in the privacy key space [40,41,42], the encryption key is secure. Likewise, except for honest $Bob_{1}$, the decryption key $\theta_{d}=2c\pi +\gamma_{1}-\theta_{1}$ is random for the remaining k−1 evaluators and attackers, since they cannot obtain any information about the decryption key. The conditional entropy of the decryption key $\theta_{d}$ is the same as its total entropy, $H\left(\theta_{d}|\;\;\;\Omega \;\;\;\right)=H\left(\theta_{d}\right)$, and then the mutual information $I\left(\theta_{d};\;\;\;\Omega \;\;\;\right)=H\left(\theta_{d}\right)-H\left(\theta_{d}|\;\;\;\Omega \;\;\;\right)=0$. So, the decryption key $\theta_{d}$ is also secure. □

(2) **The security of the ciphertext quantum state sequences during transmission**

During the encryption and evaluation computation stages, the transmitted ciphertext quantum state sequences could be subject to an intercept–resend attack by an eavesdropper, Eve, over the quantum channel. If Eve intercepts and resends the quantum states, it may alter the state of the transmitted ciphertexts, potentially leading to incorrect quantum computations by the evaluators on incorrect ciphertext quantum states and erroneous decryption by the data owner. To defend against this, we insert *l* decoy particles into the transmitted ciphertext quantum state sequence. These particles are randomly placed and can take one of four possible states {|1〉,|0〉,|+〉,|−〉}. If Eve intercepts and measures a decoy particle, the probability of correctly choosing the measurement basis is 1/2, and the probability of matching the correct basis is also 1/2.The probability that Eve successfully intercepts the decoy particles without altering their state is ${\left(\frac{l}{4(m+l)}\right)}^{l}$ where *m* is the length of the ciphertext sequence and *l* is the number of decoy particles inserted. The probability that Eve’s attack is undetected is

(24) $$Pr_{sd}=1-{\left(\frac{l}{4(m+l)}\right)}^{l}.$$

When the number *l* of inserted decoy particles in the transmitted quantum state sequence is sufficiently large, the probability $Pr_{sd}$ of detecting the intercept–resend attack approaches 1, ensuring the security of the transmitted ciphertext quantum state sequence. Thus, by inserting decoy particles with different states, we can effectively prevent intercept–resend attacks. The ciphertext quantum state sequences is secure during transmission.

(3) **The security of the plaintext quantum state sequence**

**Theorem 2.**  *The proposed TQHE scheme is q-IND-CPA secure. For any OPT adversary A, he cannot distinguish between the original ciphertext quantum state sequences encrypted from different original plaintext quantum state sequences.*

**Proof.**  We prove Theorem 2 through the following indistinguishability game $Game_{A,\xi }$. For any adversary A and a security parameter κ, the proposed TQHE scheme holds that

(25) $$\mathrm{Pr}\left[Game_{A,\xi }\left(\kappa \right)=1\right]\leq \frac{1}{2}+negl\left(\kappa \right),$$

where $\mathrm{Pr}\left[Game_{A,\xi }\left(\kappa \right)=1\right]$ is the probability that A wins the indistinguishability game $Game_{A,\xi }$ and negl(κ) is a negligible function. In the game $Game_{A,\xi }$, an adversary $A=(A_{1},A_{2})$ is constructed. $A_{1}$ firstly selects an input $|m_{0}\rangle$ according to the evaluation key $\rho_{evk_{i}}$. The challenger samples a random bit r∈{0,1}. If r=1, the input $|m_{0}\rangle$ is encrypted to $|c_{a}\rangle$ and send to $A_{2}$; otherwise, r=0, $|m_{0}\rangle$ is swapped out and replaced by a dummy input |0〉〈0| to obtain $|c_{a}\rangle$. Then, the challenger sends the challenge ciphertext $|c_{a}\rangle$ to $A_{2}$. In our proposed TQHE scheme, ${\mathrm{Adv}}_{E}^{q\text{-}\mathrm{IND}\text{-}\mathrm{CPA}}\left(A\right)$ is the advantage of winning the q-IND-CPA game when the adversary A faces the encryption algorithm, where ${\mathrm{Adv}}_{E}^{q\text{-}\mathrm{IND}\text{-}\mathrm{CPA}}\left(A\right)\leq negl\left(\kappa \right)$. The probability that $A_{2}$ guesses $r^{\prime }=r$ (wins the $Game_{A,\xi }$) is

(26) $$\mathrm{Pr}\left[Game_{A,\xi }\left(\kappa \right)=1\right]=\mathrm{Pr}\left[r^{\prime }=r\right]=\frac{1}{2}+{\mathrm{Adv}}_{E}^{q\text{-}\mathrm{IND}\text{-}\mathrm{CPA}}\left(A\right)\leq \frac{1}{2}+negl\left(\kappa \right),$$

we can conclude that the adversary A wins the indistinguishability game with a probability no higher than 1/2. Therefore, the proposed TQHE scheme satisfies q-IND-CPA. In other words, the plaintext quantum state sequence is secure. □

(4) **The security of the result after computations on the plaintext quantum state sequence**

**Theorem 3.**  *The proposed TQHE scheme for an arbitrary OPT adversary A, he cannot distinguish the final ciphertext quantum state sequences evaluated computations on different original plaintext quantum state sequences.*

**Proof.**  We prove Theorem 3 through the following indistinguishability game $Game_{A,\eta }$. In the game $Game_{A,\eta }$, an adversary $A=(A_{1},A_{2}$ is constructed. $A_{1}$ firstly selects an input $|m_{0}\rangle$ according to the evaluation key $\rho_{evk_{i}}$. The challenger samples a random bit r∈{0,1}. If r=1, the input $|m_{0}\rangle$ is encrypted to $|c_{a}\rangle$ and sent to $A_{2}$; otherwise, r=0, $|m_{0}\rangle$ is swapped out and replaced by a dummy input |0〉〈0| to obtain $|c_{a}\rangle$. Then, the challenger computes the homomorphic evaluation $\mathrm{TQHE}.\mathrm{Eval}\;(\chi_{i},\;\rho_{evk_{i}},\;|c_{a}\rangle )$ to obtain the result $|c_{f}\rangle$. These two types of computations are represented by η.real and η.ideal, respectively. Then, the challenger decrypts the $|c_{f}\rangle$ to obtain $|m_{f}\rangle$ which is sent to $A_{2}$. In our proposed TQHE scheme, $A_{2}$ guesses $r^{\prime }=r$ with a probability significantly lower than 1/2. The probability that the adversary A wins is

(27) $$\begin{array}{cc} \mathrm{Pr}\left[Game_{A,\eta }\left(\kappa \right)=1\right] & =\frac{1}{2}\mathrm{Pr}\left[\eta .real\right]+\frac{1}{2}\mathrm{Pr}\left[\eta .ideal\right]\\ \; & =\mathrm{Pr}\left[r=0\right]\mathrm{Pr}\left[A_{3}\;\mathrm{guesses}\;0\;\;\;|\;\;\;r=0\right]+\mathrm{Pr}\left[r=1\right]\mathrm{Pr}\left[A_{3}\;\mathrm{guesses}\;1\;\;\;|\;\;\;r=1\right]\\ \; & \leq \frac{1}{2}+negl\left(\kappa \right). \end{array}$$

□

We can conclude that A wins the indistinguishability game with a probability no higher than 1/2. That means the adversary A cannot distinguish the final ciphertext quantum state sequences by evaluating computations on different original plaintext quantum state sequences. Furthermore, the result after computations on the plaintext quantum state sequence is secure.

## 6. Comparisons

In this section, the approximate efficiency of the scheme will be analysed in terms of the approximate counts of the total computational complexity and time complexity of one successful execution of the TQHE scheme by the data owner and the evaluators. Comparison with the existing TQHE schemes [31,32] in terms of flexibility and efficiency shows that our proposed TQHE scheme has better flexibility and higher efficiency.

**Computational complexity:** In the key generation stage, the data owner computes and distributes the key information for *k* evaluators with complexity O(t+n+k); in the encryption sharing stage, the data owner performs *m* encryption unitary operations and the evaluators perform km phase shift unitary operations with complexity O(m); in the evaluation computation stage, *k* evaluators perform the combined execution of the homomorphic evaluation computation task with complexity O(Cm) (where *C* is the total number of evaluation computation gates to be performed, a non-negligible constant); in the decryption stage, the data owner performs *m* decryption unitary operations with complexity O(m). Hence, the computational complexity of the proposed scheme is O(t+n+k), O(m), O(Cm), O(m).

**Time complexity:** In the single execution scheme, in the key generation phase, the data owner executes the key generation and distribution algorithm with complexity O(t+n+k); in the encryption sharing stage, the data owner applies *m* encryption unitary operations, while the evaluators execute km phase shift unitary operations, with an overall complexity of O(m); in the evaluation computation stage, *k* evaluators cooperate to perform all homomorphic evaluation computation tasks with complexity O(tk) (*t* is the average computation time for each evaluator to perform the assigned evaluation task); in the decryption stage, the data owner performs *m* decryption unitary operations with complexity O(m). Therefore, the computational complexity of the proposed scheme is O(t+n+k), O(m), O(tk), O(m).

As shown in Table 2, the total computational complexity and time complexity of the TQHE schemes of Chen et al. [31] and Liu et al. [32] are the same in the four stages of the TQHE scheme, which are $O(n^{k}\cdot k^{3})$, O(m), O(Cm), O(m) and $O(n^{k}\cdot k^{3})$, O(m), O(tk), O(m), respectively. Moreover, in our scheme, any evaluators have the ability to perform any single-qubit unitary operations from {X,Y,Z,H,S,T,U(θ)} and can execute any single-qubit gate unitary operations from $\left\{X,\;Y,\;Z,\;H,\;S,\;T\right\}$, which shows that our proposed scheme has more excellent flexibility compared to scheme [31] and scheme [32].

## 7. Conclusions

In summary, we first propose a novel TQHE network scheme with a flexible number of evaluators in this paper, in which any evaluators have the ability to perform single-qubit unitary operations {X,Y,Z,H,S,T,U(θ)} and can execute any single-qubit gate unitary operations from the set of single-qubit gates {X,Y,Z,H,S,T} assigned by the data owner on the ciphertext quantum state sequence. Subsequently, a specific (3, 5)-threshold QHE example is given to further show the correctness and feasibility of our scheme. In addition, the example is then simulated on the IBM quantum computing cloud platform, and the results of the experiment also verify the correctness and feasibility of the scheme. Finally, a comprehensive analysis of the security of the encryption/decryption private keys, ciphertext quantum state sequences during transmission, the plaintext quantum state sequence, and the result after computations on the plaintext quantum state sequence indicates that our proposed scheme is secure.

## Figures and Tables

**Table 1:** Comparison of experimental results using two different backends and different shots for the example.

Running Environment	Result (a)	Result (b)	Result (c)	Result (d)	Result (e)
Backend1: ibm_brisbane	|0〉: 99.8%	|0〉: 99.3%	|0〉: 99.6%	|0〉: 99.4%	|0〉: 99.6%
Shots: 1024	|1〉: 0.2%	|1〉: 0.7%	|1〉: 0.4%	|1〉: 0.6%	|1〉: 0.4%
Backend1: ibm_brisbane	|0〉: 99.8%	|0〉: 99.5%	|0〉: 99.4%	|0〉: 99.9%	|0〉: 99.7%
Shots: 4096	|1〉: 0.2%	|1〉: 0.5%	|1〉: 0.6%	|1〉: 0.1%	|1〉: 0.3%
Backend1: ibm_brisbane	|0〉: 99.9%	|0〉: 99.5%	|0〉: 99.2%	|0〉: 99.7%	|0〉: 99.8%
Shots: 8192	|1〉: 0.1%	|1〉: 0.5%	|1〉: 0.8%	|1〉: 0.3%	|1〉: 0.2%
Backend2: ibm_lagos	|0〉: 99.6%	|0〉: 99.8%	|0〉: 99.3%	|0〉: 99.5%	|0〉: 99.4%
Shots: 1024	|1〉: 0.4%	|1〉: 0.2%	|1〉: 0.7%	|1〉: 0.5%	|1〉: 0.6%
Backend2: ibm_lagos	|0〉: 99.9%	|0〉: 99.5%	|0〉: 99.6%	|0〉: 99.4%	|0〉: 99.5%
Shots: 4096	|1〉: 0.1%	|1〉: 0.5%	|1〉: 0.4%	|1〉: 0.6%	|1〉: 0.5%
Backend2: ibm_lagos	|0〉: 99.8%	|0〉: 99.7%	|0〉: 99.9%	|0〉: 99.4%	|0〉: 99.7%
Shots: 8192	|1〉: 0.2%	|1〉: 0.3%	|1〉: 0.1%	|1〉: 0.6%	|1〉: 0.3%

**Table 2:** Comparison with the schemes proposed by Chen et al. [31] and by Liu et al. [32].

Property	Chen et al.’s Scheme [31]	Liu et al.’s Scheme [32]	The Proposed Scheme
The scope of quantum evaluation computation of *k* evaluators	${\left\{X,Y,Z,H\right\}}^{1}$	${\left\{X,Y,Z,H,S,T\right\}}^{1}$	${\left\{X,Y,Z,H,S,T\right\}}^{1}$
	∗${\left\{X,Y,Z,H\right\}}^{2}$	$*{\left\{X,Y,Z,H,S,T\right\}}^{2}$	$*{\left\{X,Y,Z,H,S,T\right\}}^{2}$
	∗⋯	∗⋯	∗⋯
	$*{\left\{X,Y,Z,H\right\}}^{k-1}$	$*{\left\{X,Y,Z,H,S,T\right\}}^{k-1}$	$*{\left\{X,Y,Z,H,S,T\right\}}^{k-1}$
	$*{\left\{X,Y,Z,H,S,T\right\}}^{k}$	$*{\left\{X,Y,Z,H,S,T\right\}}^{k}$	$*{\left\{X,Y,Z,H,S,T\right\}}^{k}$
The quantum computing capability possessed by evaluators		The first k−1 evaluators:	
	All *k* evaluators:	{X,Y,Z,U(θ)}	All *k* evaluators:
	{X,Y,Z,H,S,T,U(θ)}	The *k*th evaluators:	{X,Y,Z,H,S,T,U(θ)}
		{X,Y,Z,H,S,T,U(θ)}	
Computational complexity	$O(n^{k}\cdot k^{3})$,O(m),O(Cm), O(m)	$O(n^{k}\cdot k^{3})$,O(m),O(Cm), O(m)	O(t+n+k), O(m), O(Cm), O(m)
Time complexity	$O(n^{k}\cdot k^{3})$,O(m),O(tk), O(m)	$O(n^{k}\cdot k^{3})$,O(m),O(tk), O(m)	O(t+n+k), O(m), O(tk), O(m)

“∗” represents the connection of the quantum evaluation computation scopes of each evaluator.

## Data Availability

The original contributions presented in the study are included in the article; further inquiries can be directed to the corresponding author.
